# Interaction of acetic acid bacteria and lactic acid bacteria in multispecies solid-state fermentation of traditional Chinese cereal vinegar

**DOI:** 10.3389/fmicb.2022.964855

**Published:** 2022-09-29

**Authors:** Menglei Xia, Xiaofeng Zhang, Yun Xiao, Qing Sheng, Linna Tu, Fusheng Chen, Yufeng Yan, Yu Zheng, Min Wang

**Affiliations:** ^1^State Key Laboratory of Food Nutrition and Safety, Key Laboratory of Industrial Fermentation Microbiology, Ministry of Education, College of Biotechnology, Tianjin University of Science and Technology, Tianjin, China; ^2^Hubei International Scientific and Technological Cooperation Base of Traditional Fermented Foods, Huazhong Agricultural University, Wuhan, China; ^3^Shanxi Zilin Vinegar Industry Co., Ltd., Shanxi Province Key Laboratory of Vinegar Fermentation Science and Engineering, Taiyuan, China

**Keywords:** Chinese cereal vinegar, acetic acid bacteria, lactic acid bacteria, microbial interaction, amensalism, *Acetobacter pasteurianus*, *Lactobacillus helveticus*

## Abstract

The microbial community plays an important role on the solid-state fermentation (SSF) of Chinese cereal vinegar, where acetic acid bacteria (AAB) and lactic acid bacteria (LAB) are the dominant bacteria. In this study, the top-down (*in situ*) and bottom-up (*in vitro*) approaches were employed to reveal the interaction of AAB and LAB in SSF of Shanxi aged vinegar (SAV). The results of high-throughput sequencing indicates that *Acetobacter pasteurianus* and *Lactobacillus helveticus* are the predominant species of AAB and LAB, respectively, and they showed negative interrelationship during the fermentation. *A. pasteurianus* CGMCC 3089 and *L. helveticus* CGMCC 12062, both of which were isolated from fermentation of SAV, showed no nutritional competition when they were co-cultured *in vitro*. However, the growth and metabolism of *L. helveticus* CGMCC 12062 were inhibited during SSF due to the presence of *A. pasteurianus* CGMCC 3089, indicating an amensalism phenomenon between these two species. The transcriptomic results shows that there are 831 differentially expressed genes (|log2 (Fold Change)| > 1 and, *p* ≤ 0.05) in *L. helveticus* CGMCC 12062 under co-culture condition comparing to its mono-culture, which are mainly classified into Gene Ontology classification of molecular function, biological process, and cell composition. Of those 831 differentially expressed genes, 202 genes are up-regulated and 629 genes are down-regulated. The down-regulated genes were enriched in KEGG pathways of sugar, amino acid, purine, and pyrimidine metabolism. The transcriptomic results for *A. pasteurianus* CGMCC 3089 under co-culture condition reveals 529 differentially expressed genes with 393 up-regulated and 136 down-regulated, and the genes within KEGG pathways of sugar, amino acid, purine, and pyrimidine metabolism are up-regulated. Results indicate an amensalism relationship in co-culture of *A. pasteurianus* and *L. helveticus*. Therefore, this work gives a whole insight on the interaction between the predominant species in SSF of cereal vinegar from nutrient utilization, endogenous factors inhibition and the regulation of gene transcription.

## Introduction

Fermentation is one of the oldest technologies for food preservation, moreover, it allows foods to achieve better flavor and functions (Tamang et al., 2016). Many traditionally fermented foods are typically produced by a naturally enriched microbiota, such as sourdough, sausages, cheese and vinegar (Kleerebezem and van Loosdrecht, 2007; Smid and Lacroix, 2013). Regardless of the raw materials, these consortia microbes contribute to the desired characteristics of the final product through biological processes (Papalexandratou et al., 2011; Lu et al., 2018; Yunita and Dodd, 2018). Compared with mono-culture, co-culture is more conducive to the production of complex metabolites. Under co-cultivation conditions, the yeasts provide various nutrients such as amino acids, unsaturated fatty acids and vitamins for the growth of lactic acid bacteria (LAB; Furukawa et al., 2013). In general, the fermentation process conducted by natural microbial consortia is more flexible and robust than that with single microorganism, even providing greater resistance to bacteriophage attack (Smid and Lacroix, 2013).

In China, many famous vinegars are produced with a spontaneous solid-state fermentation (SSF) technology with cereals as the main raw material, including Shanxi aged vinegar (SAV), Sichuan bran vinegar, Zhenjiang aroma vinegar, and Duliu mature vinegar, which have a history of thousands of years. Due to the open fermentation technology, multiple microorganisms including molds, yeasts, and bacteria co-exist in the SSF process (Wu et al., 2021a). Studies have shown that LAB and acetic acid bacteria (AAB) are the two dominant bacteria in the SSF process (Nie et al., 2013; Wu et al., 2021b; Huang et al., 2022a). Lactic acid and acetic acid are the most important organic acids in vinegar, accounting for more than 90% of the total acid, which are produced by LAB and AAB (Wu et al., 2021b). The most common LAB and AAB in cereal vinegar fermentation are *Lactobacillus helveticus* and *Acetobacter pasteurianus* (Huang et al., 2022b).

Microbe-microbe interactions are widespread in the multispecies food fermentation. The coexistence of microorganisms in the same ecological niche can lead to positive or negative interactions, affecting their growth patterns, adaptation, and ability to synthesize proteins and metabolites (Bertrand et al., 2014; Feng et al., 2018; Nai and Meyer, 2018). During the fermentation process of Fukuyama pot vinegar (liquid-state fermentation), LAB and yeasts are mutually beneficial (Furukawa et al., 2013). Due to the aerobic growth characteristic, AAB generally grow in the upper medium with rich oxygen content, providing a suitable low-oxygen environment for yeast and LAB (Furukawa et al., 2013). In cocoa pulp fermentation lactic acid that will inhibit the growth of LAB in high concentration can be utilized by AAB to produce acetoin (Adler et al., 2014). Different from some liquid-state fermentation, in SSF system the mass (e.g., oxygen and water) and bio-heat transfer is poor, resulting the growth inhibition due to the endogenous environmental factors (Zhang et al., 2020). Moreover, these solid substrates (including wheat bran, sorghum and rice hull) can serve as supports (carriers) for the microbial cells, and the immobilized microorganism might be help to improve the survival of microorganism under stress conditions (Oleinikova et al., 2020). The high niche overlap, nutritional deficiency and optimal redox potential of microorganisms are important factors affecting the symbiosis of microorganisms (Edwardson and Hollibaugh, 2018). However, the interaction mechanism of the predominant microorganism in SSF of Chinese cereal vinegar is not clear yet. Due to the significant complementarity, the top-down microbiomics method and the bottom-up rational analysis are usually adopted to study the interactions between microorganisms (Wolfe et al., 2014; Liang et al., 2016; Lu et al., 2016; Zheng et al., 2018). In this study, top-down (*in situ*) and bottom-up (*in vitro*) approaches were applied to reveal the interaction between LAB and AAB in SSF of cereal vinegar.

## Materials and methods

### Samples

Samples of *Cupei* (brewing mash of Chinese cereal vinegar with solid-state fermentation technology) used in this study were collected from a SAV factory (Qingxu, China) on day 0, 1, 3, 5 and 7 during the fermentation process. A five-point sampling method was used to collect the samples. They were parallelly collected from three fermenter. These samples were analyzed according to previous methods (Nie et al., 2013).

### Strains and media

Strains *A. pasteurianus* CGMCC 3089 and *L. helveticus* CGMCC 12062 were used in this study, which were isolated from the SAV fermentation of the same factory and were registered in Chinese General Microbiological Culture Collection Center. GY and MRS media were applied for *A. pasteurianus* and *L. helveticus* culture, respectively. SSF medium (Solid-state fermentation medium, SSFM) was used for *in vitro* solid-state fermentation, which consists of the following ingredients: wheat bran, 30 g/100 g; rice husk, 10 g/100 g; MRS (de Man, Rogosa, Sharp) medium, 55 g/100 g, ethanol, 4 g/100 g; acetic acid, 0.5 g/100; and lactic acid, 0.5 g/100. The *in vitro* SSF was performed in a 5,000 ml ceramic pot containing 3,000 g of SSFM.

### *In situ* analysis of LAB and AAB communities

The metagenomic DNA of *Cupei* was extracted in accordance with a previously described method (Nie et al., 2013). The V3-V4 region of 16S rDNA was amplified with the metagenome as template. The result fragment was sent to GENEWIZ Company (Suzhou, China) for sequencing analysis using the Illumina MiSeq platform. Sequencing reads were sorted to specific samples on the basis of unique barcodes, which were subsequently trimmed. Sequencing reads were removed if the reads were <200 or >1,000 bp, had a non-exact barcode match, exceeded two ambiguous bases, exceeded eight consecutive identical bases, or had average quality scores <25. A nearest alignment space termination-based sequence aligner was used to align sequences to a custom reference based on SILVA alignment. Chimeric sequences were identified and filtered by quality. Chimera-free sequences were clustered in operational taxonomic units (OTUs) defined by 97% similarity by using a complete-linkage clustering tool (Gan et al., 2017). Representative sequences per OTU were classified in accordance with previously described methods, and the LAB and AAB communities were obtained.

CCA (Canonical Correspondence Analysis) was performed to determine the species-species relationship between LAB and AAB community and fermentation parameters (pH, total acid, reducing sugar, and temperature, and metabolites ethanol, lactic acid, and acetic acid). The analysis was conducted using Canoco for Windows v4.51 and Canodraw (Wageningen UR, Netherlands). The interaction network of the microorganisms *Lactobacillus* and *Acetobacter* was inferred using Metagenomic Microbial Interaction Simulator (MetaMIS) software (Shaw et al., 2016). The dynamic abundance of OTUs that were identified as *Acetobacter* and *Lactobacillus* were used for MetaMIS analysis. The website address for obtaining MetaMIS software is.^1^ Data standardization was performed using IBM SPSS 19.0.^2^

### *In vitro* solid-state fermentation of *Acetobacter pasteurianus* and *Lactobacillus helveticus*

*Acetobacter pasteurianus* CGMCC 3089 was inoculated into GY medium (100 ml) and incubated aerobically at 30°C for 24 h in a rotary shaker (180 r/min). *L. helveticus* CGMCC 12062 was statically incubated in MRS medium (100 ml) at 37°C for 24 h. The *in vitro* SSF was performed by inoculating them individually (mono-culture) or together (co-culture) into the SSFM with the initial count of 10^7^ CFU/g, which was the same as their initial count in the SSF of SAV (Zheng et al., 2022). The *in vitro* SSF was performed at 35°C, and the medium was stirred every 12 h to improve the mass transfer. Therefore, the time curves in mono-culture and co-culture of *L. helveticus* and *A. pasteurianus* were produced.

Samples of mono-culture at 24 h and co-culture at 24 h with SSFM were collected for transcriptomic analysis. In brief, 5.0 g of SSFM sample was added into 95 ml of DEPC water and shaken for 5 min. The samples were filtered with two layers of sterile cheesecloth, and the filtrate was centrifuged at 8,000× *g* for 10 min at 4°C. The pellet was then subjected to RNA extraction using the RNA plus Kit (Takara Biotechnology, Dalian, China) following manufacturer’s procedure. Transcriptome sequencing was performed by Novogene Co., Ltd. (Tianjin). The transcriptome sequencing data has been uploaded to NCBI Short Read Archive and the accession number is PRJNA833267. The genomes of strain *A. pasteurianus* IFO3283-01 (PRJDA31129) and *L. helveticus* CGMCC 1.1877 (PRJNA34619) were used as the references to identify the genes of transcriptome. Gene expression level was calculated by fragment per kilo bases per million reads (FPKM) using RSEM software (v1.2.6) with 0.1 as the rounding threshold for gene expression. The GI numbers of the selected genes were imported into the Kyoto Encyclopedia of Genes and Genomes (KEGG) database^3^ for biological pathway analysis. A differential metabolic network was constructed based on transcriptomic analysis, mainly involving glycolytic pathway, TCA cycle, ABC transport system, amino acid metabolism, purine and pyrimidine metabolism, and two-component system.

Several representative genes were selected to analyze the potential interaction mechanism by using the method of qRT-PCR. Software of Primer 5 was used for primers design, which is listed in Table 1. The total RNA was isolated using RNA Plus Kit as mentioned above. To remove residual DNA, total RNA was treated with DNase I for 30 min at 37°C. RNA samples were reverse transcribed with Revert Aid™ First Strand cDNA Synthesis Kit (Takara Biotechnology, Dalian, China) in accordance with the manufacturer’s instructions. The quantity analysis of RNA was performed with ABI Step one system (Applied Biosystems, United States). The results were expressed by using 2^−△△Ct^ with the 16S rRNA as the internal standard gene. For each gene, the relative transcription of mono-culture was defined as the expression level of 1.0, and the result was expressed as the fold increased in mRNA compared with the control sample (log2).

**Table 1:** Primer used for qRT-PCR analysis of genes transcription in *Lactobacillus helveticus.*

Primer	Sequence (5′-3′)	Corresponding genes
LAF_RS00730_F	TTTAAGGTCCATGGCTTTGC	Aspartate aminotransferase
LAF_RS00730_R	CTCGTCGTCTTGTTCCAGGT	
LAF_RS09565_F	GTAACATGGTCGGGAACTGG	ABC transporter ATP-binding protein
LAF_RS09565_R	GGGGTCGGTAAGTCAACCTT	
LAF_RS04525_F	GGTGAAGGAAGTGGTCATCG	3-Phosphoglycerate dehydrogenase
LAF_RS04525_R	GTAGCCAATCACGTCCATCC	
LAF_RS07750_F	ATCAGGCAGCAGTTGGTTTC	Succinate-semialdehyde dehydrogenase
LAF_RS07750_R	AAGGTCTTCAACACCCAACG	
LAF_RS10410_F	TTAACGATCGCCTTGGAAAC	Alcohol dehydrogenase
LAF_RS10410_R	CCTTCATCAACCCGTACACC	
LAF_RS04455_F	GCCGACCAGTTTGAGTTCAT	ATP phosphoribosyl transferase
LAF_RS04455_R	GGGTCAAAGTCTTCGGTTGA	
16S F	AGCGAGCAGAACCAGCAGATT	16S rRNA
16S R	TGCACCGCGGGGCCATCCCA	

### The effects of endogenous factors on cell growth in co-culture

To analyze the effects of endogenous factors on cell growth, the SSF medium without ethanol and acids was prepared. And then the initial concentrations of ethanol, lactic acid, and acetic acid were set as 0, 1.5, 3.0, 4.5 g/100 g, 0, 1.0, 2.0, 3.0 g/100 g, and 0, 1.0, 2.0, 3.0 g/100 g, respectively. The fermentation was performed at 35°C. In particular, to compare the effect of temperature on cell growth the SSF medium without ethanol and acids was used, and the fermentation was conducted at temperatures of 30°C, 37°C, and 45°C. Those endogenous factors were set according to their change in the SSF process of SAV. The effects of endogenous factors on microbial growth in co-culture condition were analyzed by comparing their CFU (Colony-Forming Unit) changes after 24 h co-culture. The colon of *L. helveticus* and *A. pasteurianus* on same plate can be distinguished by the transparent.

### Analytical methods

For the analysis of physicochemical property, 5.0 g of *Cupei* and 95.0 ml of double distilled water were mixed for 1 h in 60°C water bath. The mixture was then centrifuged for 10 min at 8,000× *g*, and the supernatant was collected for analysis. Contents of ethanol and glucose were detected by a biosensor (SBA, Shandong, China). Lactic acid and acetic acid were determined through high-performance liquid chromatography (HPLC), as described previously (Wolfe et al., 2014).

### Statistical analysis

All experiments were conducted in triplicate unless otherwise indicated. Origin 2018 and Excel were used for data analysis. Spearman coefficient (*p*) was calculated with Origin software by paired-sample two-tailed *t*-test.

## Results

### Prediction of the interaction between the dominant microorganisms during *in situ* fermentation

**Figure 1:**
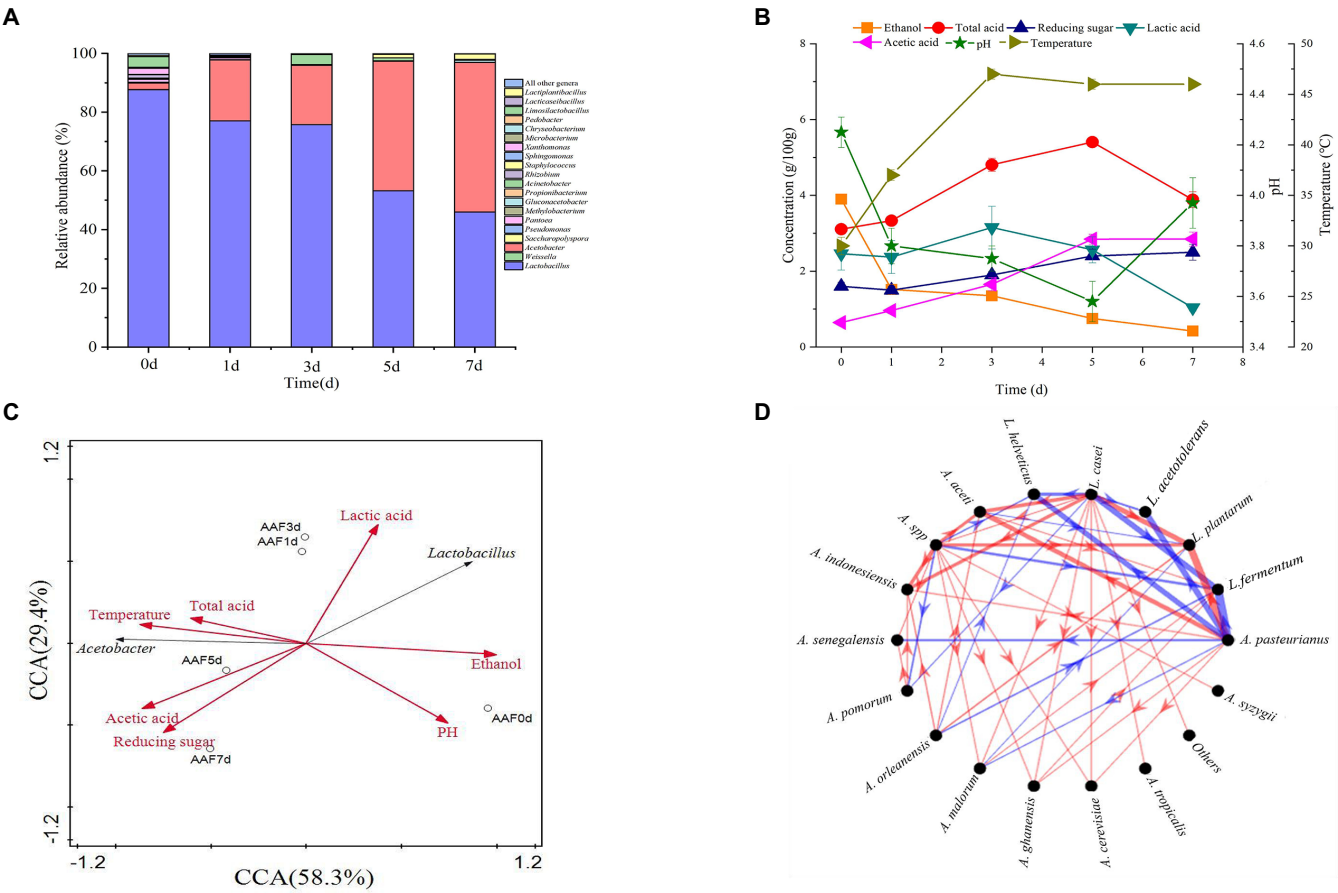
Time curves and the microbial correlations in SAV fermentation. **(A)** The composition of bacterial communities; **(B)** The physicochemical indicators; **(C)** CCA analysis; **(D)** Interaction networks of *Lactobacillus* and *Acetobacter*: Edge thickness represents the correlation value, and edge color represents the positive (red) or negative (blue) correlation.

High-throughput sequencing was used to investigate the composition and succession of bacterial communities during SSF of SAV. As shown in Figure 1A, *Lactobacillus* and *Acetobacter* are the dominant genera, accounting for more than 95% of all the bacteria. The relative abundance of *Lactobacillus* (mainly *L. helveticus* and *Lactobacillus acetotolerans*) on day 1 was 87.6%, and then gradually decreased to 46.0% on the last day of fermentation. The relative abundance of *Acetobacter* increased from 2.2 to 50.9% from day 1 to day 7. During the fermentation process the OTU with the highest relative abundance (more than 55%) was *L. helveticus*, followed with *L. acetotolerans*, *Limosilactobacillus fermentun* (*L. fermentun*), *Lacticaseibacillus casei* (*L. casei*), *Lactiplantibacillus plantarum*(*L. plantarum*; Zheng et al., 2020). 12 OTUs were classified as *Acetobacter*, including *A. pasteurianus*, *Acetobacter aceti*, *Acetobacter cerevisiae*, *Acetobacter ghanensis*, *Acetobacter indonesiensis*, *Acetobacter malorum*, *Acetobacter orleanensis*, *Acetobacter pomorum*, *Acetobacter senegalensis*, *Acetobacter senegalensis*, *Acetobacter senegalensis*, *Acetobacter* spp., among which the most dominant OTU was *A. pasteurianus* (more than 70%). LAB and AAB are the main microorganisms in SSF of SAV, however, their succession patterns differentiated. OTU of *L. plantarum* (4.34% at day 0) disappeared since day 3. The relative abundances of *L. casei* and *L. acetotolerans* decreased, while those of *L. helveticus*, *L. fermentum* increased. The relative abundance of *Acetobacter* increased. Throughout the fermentation *Lactobacillus* and *Acetobacter* were the dominant bacteria, the most OTUs were identified as *L. helveticus* and *A. pasteurianus*. Though the relative abundance of *L. helveticus* increased, its absolute number decreased due to the various stress factors (Zheng et al., 2022), indicating the growth inhibition in SSF of SAV. While, the absolute number of *A. pasteurianus* increased. Ethanol, reducing sugar, total acid, temperature, acetic acid, and lactic acid are the key physical and chemical indicators for monitoring SSF of SAV, and they also affect the cell growth and succession of microbial communities (Chen et al., 2017). As shown in Figure 1B, the ethanol content decreased from 3.89 to 0.40 g/100 g *Cupei* from day 0 to day 7. Generally, the fermentation is stopped when the total acidity no longer increases and the ethanol concentration is less than 0.5 g/100 g *Cupei*. Lactic acid increased from 2.461 to 3.152 g/100 g *Cupei* from day 0 to day 3, and then decreased to 1.039 g/100 g *Cupei* at the end of fermentation (day 7). Acetic acid accumulated from 0.646 to 2.847 g/100 g *Cupei* throughout the fermentation process. As described in Figure 1C, the abundance of *Lactobacillus* and *Acetobacter* are positively related to the content of lactic acid and acetic acid, respectively, indicating that they were the main acid producers. The temperature was highly positively related to content of *Acetobacter*, total acid, and acetic acid, and negatively related to ethanol. Those results indicated that the oxidation of ethanol to acetic acid by *Acetobacter* was the main source of bio-heat, particularly in the middle stage of fermentation (day 5 and day 7; Zhang et al., 2020). Therefore, the dynamic of dominant genera was highly related to the endogenous factors of acetic acid, ethanol, lactic acid, and temperature. This result was in agreement with previous reports that analyzed the correlations between environmental factors and the microbial community in SSF of cereal vinegar (Lu et al., 2018; Zheng et al., 2018; Fang et al., 2021).

The interaction between microorganisms plays an important role on regulating the succession of bacterial community (Wu et al., 2021a). The MetaMIS method was used to predict the interaction network among the dominant species of *Acetobacter* and *Lactobacillus* based on the high-throughput sequencing (as shown in Figure 1D). The high negative correlations are observed between *A. pasteurianus* and *L. fermentum*, *A. pasteurianus* and *L. helveticus*, and *A. pasteurianus* and *L. acetotolerans*. The high positive correlations are between *A. pasteurianus* and *L. aceti*, *A. pasteurianus* and *L. plantarum*, and *L. plantarum* and *L. case.* The negative interactions between LAB and AAB are well known (Hutchinson et al., 2019; Chai et al., 2020) and analyzed with kinetic models (Lefeber et al., 2010; Zheng et al., 2022). In particular, the most thick edges are observed on *A. pasteurianus*, representing it plays the most important role in the succession of bacterial community. Interestingly, *L. helveticus* is the other predominant species, however only one thick edge is observed, indication less effect on the succession of bacterial community. *L. helveticus* and *A. pasteurianus* are the two dominant species in SAV fermentation, and also the most abundant microorganisms in the other cereal vinegars, such as Zhenjiang aroma vinegar and Sichuan bran vinegar (Ai et al., 2019; Huang et al., 2022b). They are responsible for the formation of organics acids, acetoin, and the other flavor compounds such as esters and phenols, and are important for the fermentation and product flavor of vinegar (Fang et al., 2021). The related research of Zhenjiang aroma vinegar shows that adding *A. pasteurianus* during the acetic acid fermentation can speed up the fermentation process and increase the content of amino acids, glutamic acid, 2, 3-butanediol, and ligustrazine and other flavor substances (Wang et al., 2016). Considering their import role in the SAV fermentation, their potential interaction mechanism was studied with the bottom-up approach.

### The interaction between two dominant species during *in vitro* fermentation

**Figure 2:**
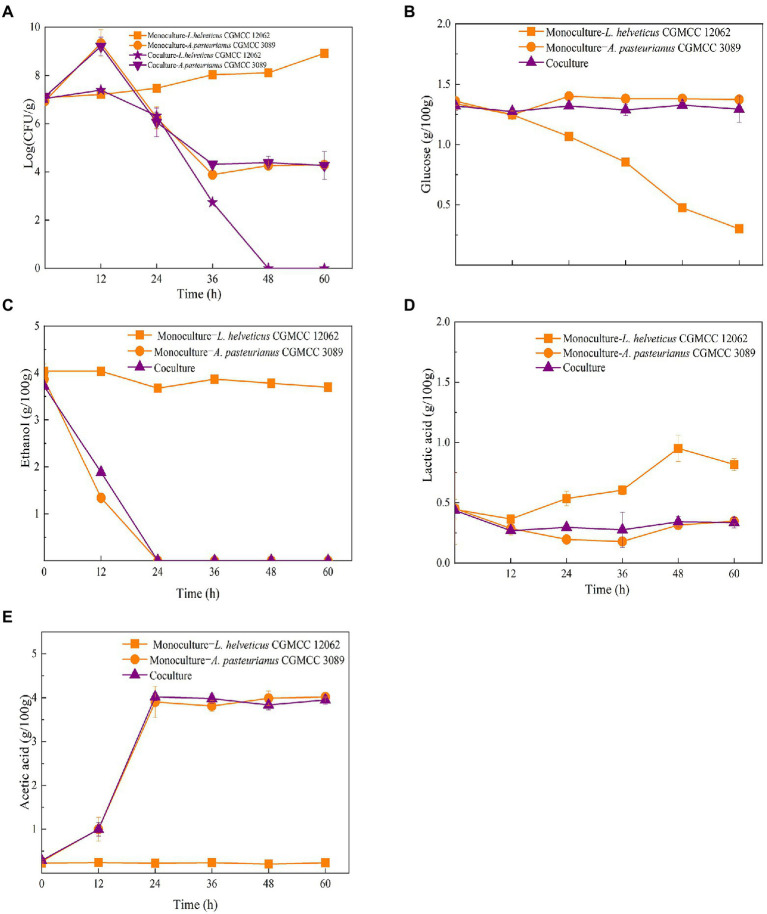
Time curves in mono-culture and co-culture of *L. helveticus* and *A. pasteurianus*. **(A)** Cell growth; **(B)** Glucose consumption; **(C)** Ethanol consumption; **(D)** Lactic acid production; **(E)** Acetic acid production.

To reveal the interaction between the two dominant species of *L. helveticus* and *A. pasteurianus* in SSF of SAV, *in vitro* SSF was performed with SSFM containing 4 g/100 g ethanol, 0.5 g/100 acetic acid, and 0.5 g/100 lactic acid, which are according to those in the initial SSF of SAV (Figure 1B). The cell growth of *A. pasteurianus* under co-culture condition was the same than in mono-culture, however, the growth of *L. helveticus* under co-culture condition was inhibited compared to that of mono-culture (Figure 2A). As described in Figure 2, under mono-culture condition ethanol was utilized as carbon sources for cell growth of *A. pasteurianus*, and the content of acetic acid increased. Glucose was utilized as carbon sources for *L. helveticus*, and the content of lactic acid increased. Those results indicate there was no nutritional competition between *A. pasteurianus* and *L. helveticus* when they were co-cultured. However, the glucose utilization and lactic acid production decreased in mono-culture (Figures 2B,D) due to the present of *A. pasteurianus*. While, the ethanol utilization, acetic acid production, and the biomass of *A. pasteurianus* were not affected by *L. helveticus* (Figures 2C,E). Adler et al. (2014) investigated the specialized metabolism of *A. pasteurianus* under cocoa pulp fermentation-simulating conditions, and found lactate was served for the biomass building blocks to maximize the cell growth and ethanol conversion.

**Figure 3:**
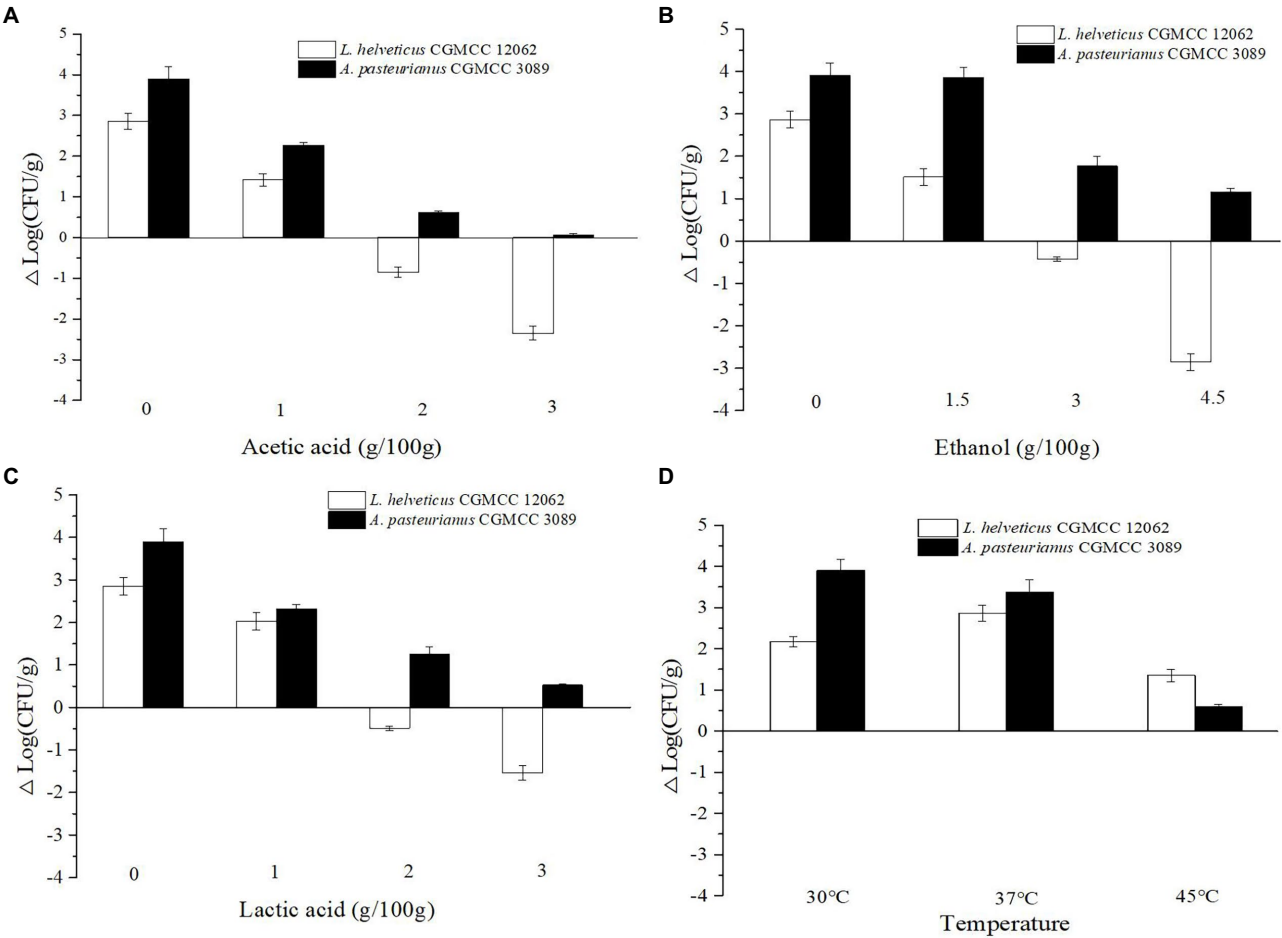
Effects of endogenous factors on microbial growth in co-culture condition. **(A)** Acetic acid; **(B)** Ethanol; **(C)** Lactic acid; **(D)** Temperature.

As shown in Figure 1C, the content of *Acetobacter* and *Lactobacillus* were highly related with the endogenous factors, ethanol, acetic acid, lactic acid, and temperature. Therefore, the effect of the endogenous factors on the cell growth was compared. As shown in Figure 3, all those endogenous factors showed an inhibitory effect on the growth of both of *A. pasteurianus* and *L. helveticus*. However, the intensity of the influence on two species was different. The cell growth of *L. helveticus* became negative when the endogenous factors, ethanol, acetic acid, lactic acid, were above 2 g/100 g acetic acid (Figure 3A), 3 g/100 g ethanol (Figure 3B), and 2 g/100 g lactic acid. While, *A. pasteurianus* was more tolerant against acetic acid (Figure 3A), ethanol (Figure 3B), and lactic acid (Figure 3C) than *L. helveticus*. Those results indicate acids were the main stress factors for *L. helveticus* growth, and *A. pasteurianus* would affect *L. helveticus* growth by producing acetic acid from ethanol and utilizing lactic acid (Adler et al., 2014). The temperature would be more than 45°C in the middle stage of SAV fermentation (Figure 1B), and *A. pasteurianus* grew better at 30°C than 37°C and 45°C 45°C (Figure 3D). The microorganism requires appropriate temperature for growth (the optimal temperature for AAB is about 30°C, and for LAB is 30–40°C). Due to the poor heat transfer efficiency in SSF systems, the temperature may influence the growth and metabolism of the microorganisms. When the temperature was above 37°C, a sharp reduction of cell growth rate was observed in *A. pasteurianus*. *L helveticus* grow better at 35–40°C than 30°C under mono-culture condition (Zhang et al., 2020). However, under 45°C its growth was inhibited (Figure 3D). Those explain how the endogenous factors affect the succession of *Acetobacter* and *Lactobacillus* during SSF of SAV. Coincidentally, all those endogenous factors are the metabolites of LAB and AAB in SAV fermentation. Thus, the *in vitro* SSF of co-culture presented an amensalism relationship between *A. pasteurianus* and *L. helveticus*. However, the interaction mechanism was not clear yet.

### Transcriptomic analysis to reveal the mechanism of interaction between *Lactobacillus helveticus* and *Acetobacter pasteurianus*

**Figure 4:**
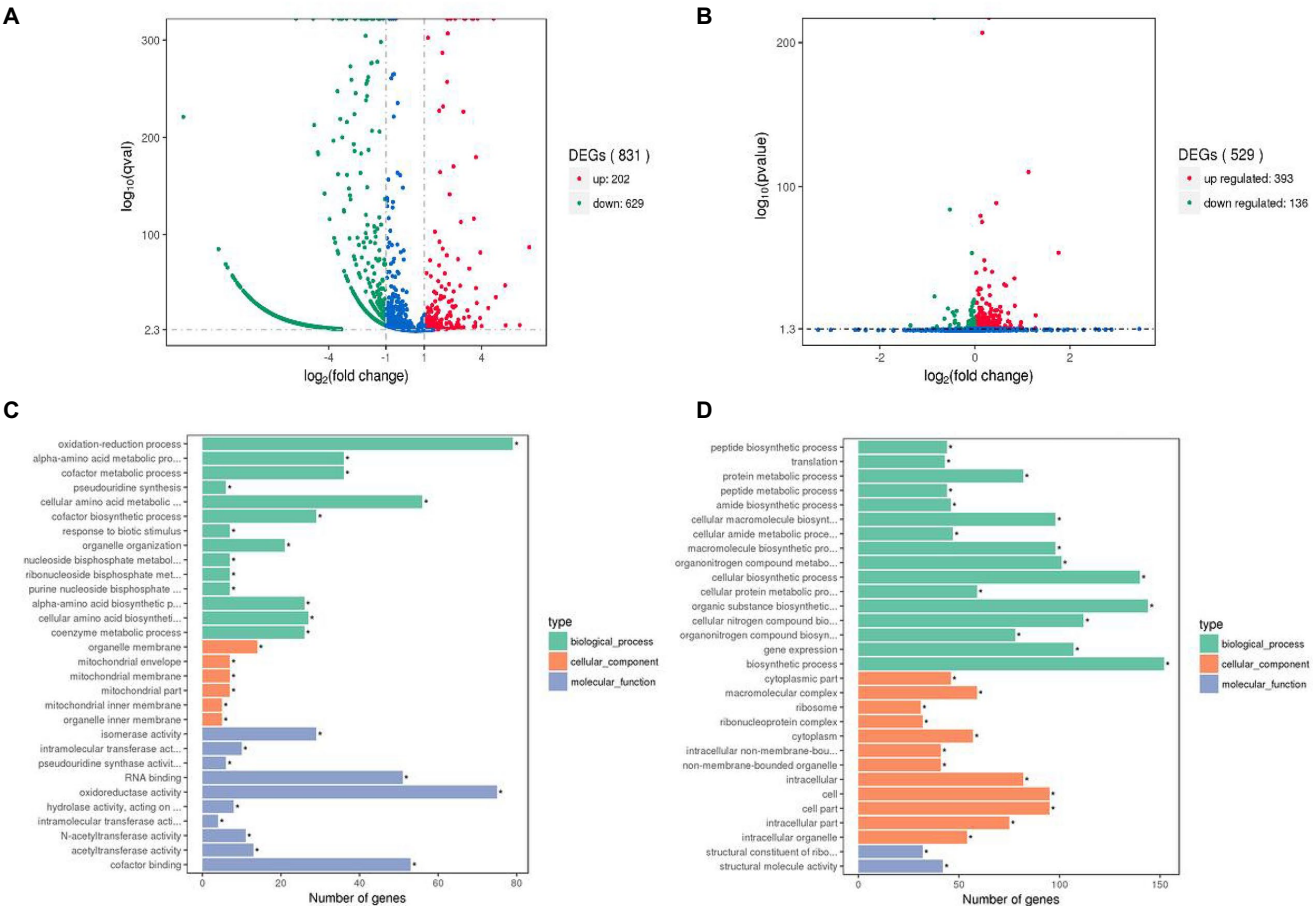
Gene expression profiles volcano map and the most enriched GO terms of differential genes. **(A)** Gene expression profiles volcano map of *L. helveticus* CGMCC 12062; **(B)** and *A. pasteurianus* CGMCC 3089; **(C)** Differential gene GO terms of *L. helveticus* CGMCC 12062; **(D)** and *A. pasteurianus* CGMCC 3089. |log2 (Fold Change)| > 1 and *p* value ≤0.05 was the criteria for transcriptome differential genes.

To reveal the potential mechanism of interaction between *L. helveticus* and *A. pasteurianus*, the transcriptomes of *L. helveticus* and *A. pasteurianus* under mono-culture and co-culture conditions were sequenced and compared. DEseq software was used to screen out statistically significant differentially expressed genes (|log2 (Fold Change)| > 1 and *p* value <0.05). The result shows that the number of differentially expressed genes in *L. helveticus* CGMCC 12062 and *A. pasteurianus* CGMCC 3089 under co-culture condition are 831 and 529, respectively, comparing to their corresponding mono-cultures (Figures 4A,B). For *L. helveticus* CGMCC 12062, 202 differentially expressed genes were up-regulated and 629 genes were down-regulated (Figure 4A), and those differentially expressed genes are mainly classified into Gene Ontology classification of molecular function, biological process, and cell composition (Figure 4C). The down-regulated genes in *L. helveticus* CGMCC 12062 were enriched in KEGG pathways of sugar, amino acid, purine, and pyrimidine metabolism. For *A. pasteurianus* CGMCC 3089, 393 differentially expressed genes were up-regulated and 136 genes were down-regulated (Figure 4B), and the genes for KEGG pathways of sugar, amino acid, purine, and pyrimidine metabolism were up-regulated (Figure 4D). There were more differentially expressed genes in *L. helveticus* under co-culture condition than those in *A. pasteurianus*. In *L. helveticus* the more genes related to cellular constituents and less genes related to molecular function were regulated than those in *A. pasteurianus*. Those results were agreed with the amensalism phenomenon between *A. pasteurianus* and *L. helveticus*.

**Figure 5:**
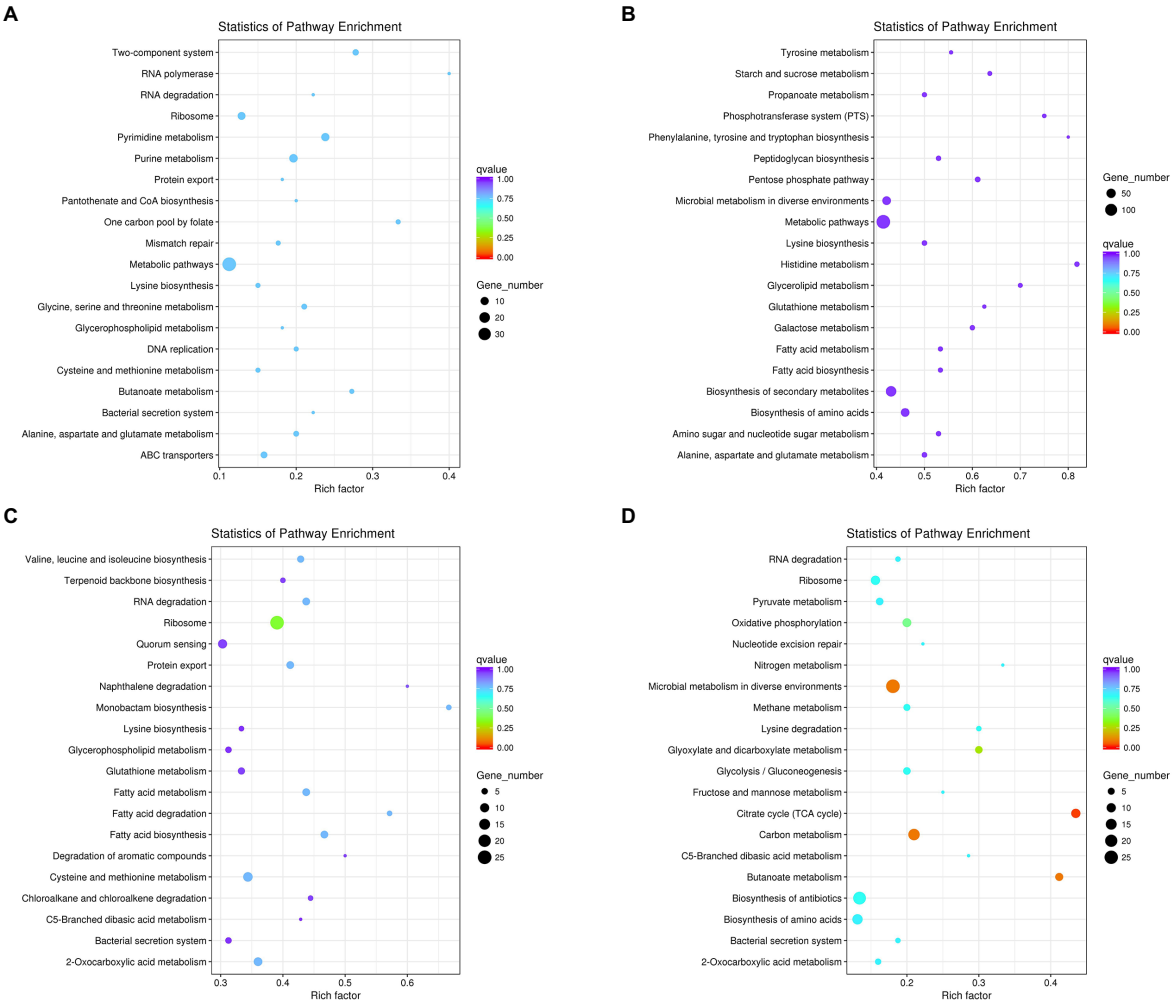
The KEGG metabolic pathway analysis of differential genes under coculture condition compared with those of mono-culture. **(A)** Pathway of up-regulated in *L. helveticus* CGMCC 12062; **(B)** and down-regulated; **(C)** Pathway of up-regulated in *A. pasteurianus* CGMCC 3089; **(D)** and down-regulated.

The KEGG metabolic pathway analysis was performed for the enrichment genes. As shown in Figure 5A, under co-culture condition the up-regulated genes in *L. helveticus* CGMCC 12062 were mainly categorized in metabolic pathway of pyrimidine, purine, amino acids (lysine, glycine, serine, cysteine, alanine, aspartate and glycine), glycerophospholipid, and energy (ABC transporters) comparing to its mono-culture condition. The genes in starch and sucrose, propanonate, amino acids (tyrosine, phenylalanine, tryptophan, histidine), galactose, and fatty acid metabolic pathways were down-regulated (Figure 5B). For *A. pasteurianus* CGMCC 3089, due to the existence of *L. helveticus* CGMCC 12062, the genes in the pathways of terpenoids, protein transport, amino acids (valine, leucine, isoleucine, lysine, cysteine, methionine), glycerophospholipids, metabolism of monocyclic β-lactams, fatty acids, aromatic compounds, hydrocarbons, sugars, and sulfur were up-regulated (Figure 5C), and those in pyruvate, glyoxylic acid, fructose and mannose pathways were down-regulated (Figure 5D).

To verify the effect of *A. pasteurianus* CGMCC 3089 on the metabolic pathways of *L. helveticus* CGMCC 12062, the transcription of several most significantly different genes, including 3-phosphoglycerate dehydrogenase, succinate-semialdehyde dehydrogenase, aspartate aminotransferase, alcohol dehydrogenase, ABC transporter ATP-binding protein, and ATP phosphoribosyl transferase, were assayed using the method of qRT-PCR. They were the important genes in pathways of EMP, TCA cycle, amino acid metabolism, acid stress tolerance, transmembrane transport, and histidine formation, respectively, and were significant differently (|log2 (Fold Change)| > 1 and *p* ≤ 0.05) descripted under mono-culture and co-culture conditions. As listed in Table 2, all those genes in *L. helveticus* CGMCC 12062 were down-regulated under co-culture condition comparing with those under mono-culture due to the presence of *A. pasteurianus* CGMCC 3089. Results from qPCR analysis were in agreement with those of transcriptomics.

**Table 2:** Assay of the transcription of genes by the method of qRT-PCR.

Genes description	log_2_ (qRT-PCR analysis)	log_2_ (RNA-seq analysis)
Aspartate aminotransferase	−8.189 ± 0.12	−8.680
ABC transporter ATP-binding protein	−6.750 ± 0.08	−7.669
3-phosphoglycerate dehydrogenase	−3.211 ± 0.13	−6.793
Succinate-semialdehyde dehydrogenase	−3.031 ± 0.04	−5.763
Alcohol dehydrogenase	−2.816 ± 0.07	−5.711
ATP phosphoribosyl transferase	−2.130 ± 0.05	−4.361

## Discussion

A lot of traditionally fermented foods are produced with a spontaneous fermentation. The dynamics of microbial community is affected by the endogenous factors, which results in the desired characteristics of the final products (Singh and Ramesh, 2008; Houngbédji et al., 2019; Zhao et al., 2020). However, the endogenous factors are complex, such as nutrients, metabolic product and environmental factors. Besides, the interrelationship among microorganisms is unavoidable in these ecosystems. Several studies have reported the interactions between microorganisms in fermentation of cereal vinegars. For example, *L. casei* and *A. pasteurianus* in Zhenjiang aromatic vinegar have synergistic effects in the synthesis of acetoin (Chai et al., 2020). In addition, other studies have shown that during the fermentation of Zhenjiang aromatic vinegar, *L. buchneri* and *L. brevis* are positively correlated, while *A. pasteurianus* and *Lactobacillus* are negatively correlated (Chai et al., 2020; Zheng et al., 2022). However, the potential mechanism of the interactions is still unclear.

In this study, the interaction of dominant microorganisms in SSF of SAV was revealed by employing top-down (*in situ*) and bottom-up (*in vitro*) approaches. Firstly, important physical and chemical indicators during *in situ* culture were monitored. Ethanol and reducing sugars are important carbon sources for AAB fermentation (Wang et al., 2015). Acetic acid and lactic acid are the most important organic acids in vinegar (Xu et al., 2011; Hen et al., 2013). The content of lactic acid increases at first and then decreases during fermentation, which is mainly due to the fact that lactic acid is used as a carbon source by other microorganisms at the later stage of fermentation (Chai et al., 2020). Temperature is the most intuitive indicator for evaluating the fermentation process of vinegar, which can reflect the metabolic activities of microorganisms (Adler et al., 2014; Li et al., 2016). Secondly, correlation analysis was performed for *Lactobacillus* and *Acetobacter* with physical and chemical indicators. In the initial stage of AAF, ethanol (the carbon source of AAB) is the main factor affecting fermentation. The main influencing factors in the middle stage of fermentation are temperature and lactic acid. In the late stage of fermentation, acetic acid become an important inhibitor of the growth of LAB, affecting the fermentation process. Thirdly, the result of MetaMIS prediction showed that *A. pasteurianus* and *L. helveticus* are inversely correlated in the SSF of SAV. Interestingly, though *L. helveticus* is the most predominant species, a few high correlations are observed from it, besides a high negative correlation with *A. pasteurianus*. Those result implies *L. helveticus* might be used for bioaugmentation of SSF of SAV to modulate the lactic acid formation without affecting the other microorganisms. Little difference was observed on growth and ethanol oxidation between mono- and co-culture of *A. pasteurianus* CGMCC 3089, while the growth and metabolism of *L. helveticus* CGMCC 12062 was significantly inhibited due to the existence of *A. pasteurianus* CGMCC 3089. In the transcriptomic analysis, the number of differentially expressed genes of *L. helveticus* CGMCC 12062 was higher than that of *A. pasteurianus* CGMCC 3089 under co-culture condition. The down-regulated genes in *L. helveticus* CGMCC 12062 were enriched in KEGG pathways of sugar, amino acid, purine, and pyrimidine metabolism, which are related to biological processes and cell compositions. These results suggest an amensalism phenomenon in co-culture of *A. pasteurianus* and *L. helveticus*.

**Figure 6:**
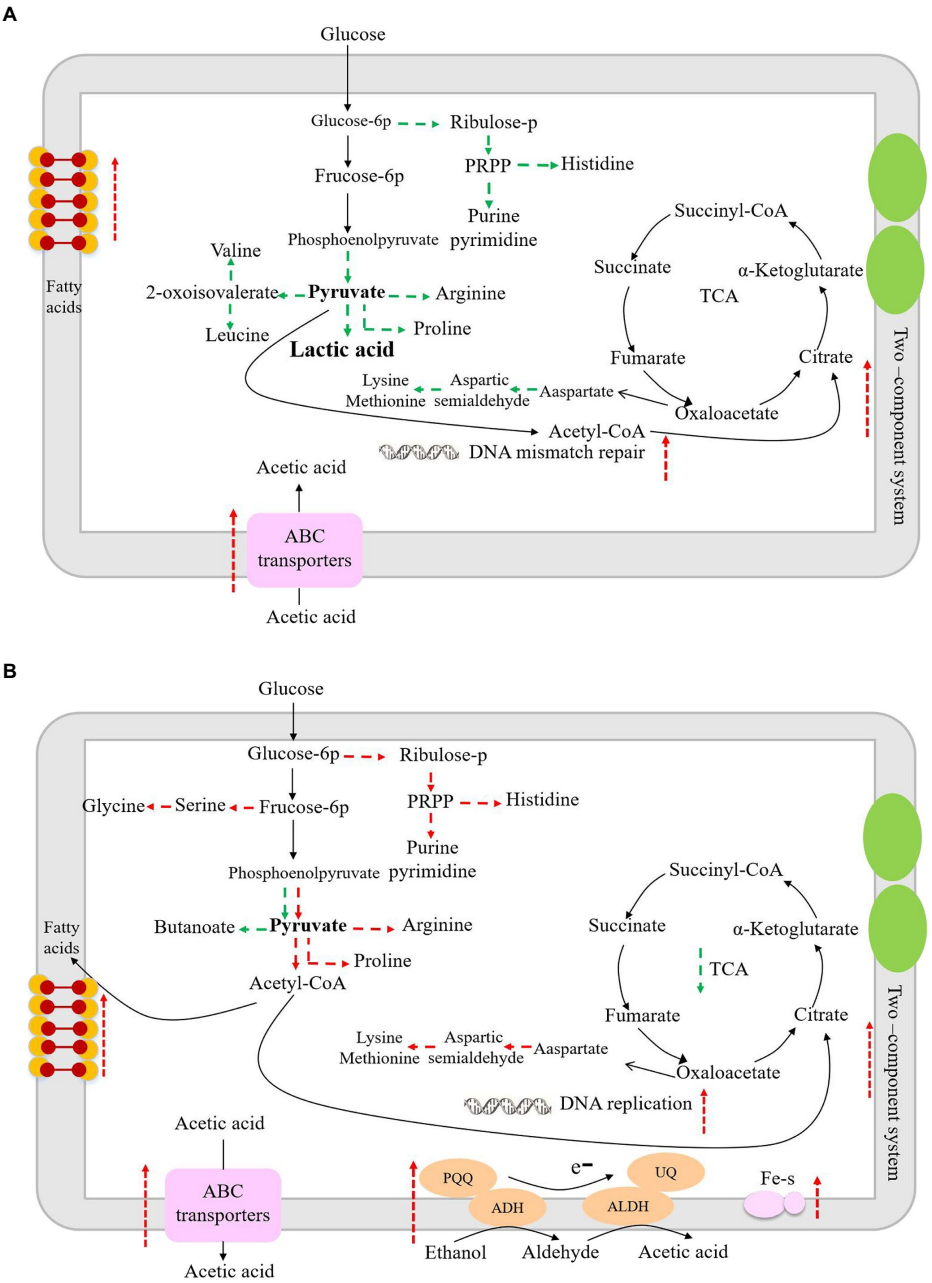
The sketch map of metabolism changes in *L. helveticus* CGMCC 12062 **(A)** and *A. pasteurianus* CGMCC 3089 **(B)** under coculture condition compared with that of mono-culture. Red represents up-regulation of metabolic pathway or gene transcription, and green represents down-regulation.

The enzyme of 3-phosphoglycerate dehydrogenase (PGDH) catalyzes the oxidation (dehydrogenation) and phosphorylation of 3-phosphoglyceraldehyde to generate 1,3-diphosphoglycerate. PGDH is an important enzyme in the glycolysis pathway and is also involved in the synthesis of serine (Zhang et al., 2017). When glucose is the sole carbon source in *Escherichia coli*, 15% of the absorbed carbon is synthesized by PGDH and other related enzymes in glycolysis, and the L-serine is then converted into other products (Pizer and Potochny, 1964). Therefore, down-regulation of PGDH may lead to decreased glucose metabolism. Succinate semialdehyde dehydrogenase oxidizes succinate semialdehyde to form succinate that then enters the tricarboxylic acid cycle. The down-regulation of succinate semialdehyde dehydrogenase indicates the decreased energy metabolism in *L. helveticus* (Legendre et al., 2020). Aspartate aminotransferase catalyzes the conversion of aspartate into oxaloacetate and glutamate, which further participate in the tricarboxylic acid cycle and glutamate decarboxylase metabolic pathways. Another study has shown that aspartate aminotransferase plays an important role in nitrogen metabolism in *Mycobacterium tuberculosis* (Jansen et al., 2020). In co-culture condition the down-regulated aspartate aminotransferase would result in the decrease of nitrogen metabolism. ABC transporters are associated with the transport of proteoglycans, amino acids, metal ions, polypeptides, proteins, and cellular metabolites (Locher, 2016). Studies have shown that LAB can change the expression of ABC transporters under acetic acid stress, which is important for bacteria to adapt to environmental stress (Kang et al., 2022). Therefore, due to the down-regulation of those enzymes, the biomass of *L. helveticus* decreased under co-cultivation condition, as shown in Figure 2. According to the main differentially expressed genes and related pathways, the speculated sketch map of metabolic changes in *L. helveticus* CGMCC 12062 (Figure 6A) and *A. pasteurianus* CGMCC 3089 (Figure 6B) under co-culture condition comparing to their corresponding mono-cultures were proposed.

SSF is one of the most features of Chinese cereal vinegar. The solid auxiliary materials, including wheat bran, rice hull, not only serve as solid supports for the cells but also provide a slow mass transfer environment. Moreover, wheat bran contains additional nutrients. In this research SSFM containing wheat bran, rice hull, MRS, and the main metabolites of predominant microorganism was used to simulate the SSF of SAV. The correlation between *A. pasteurianus* and *L. helveticus* from *in vitro* fermentation were agreement with that *in situ* fermentation. However, comparing to the real SSF process, a lot of substrates are missed, e.g., some sugars, esters, which might affect the cell growth. To simulate the real SSF of cereal vinegar assembling wheat bran, rice hull and the sterilized leach liquor of *Cupei* might be a potential candidate medium.

## Conclusion

In this study, top-down (*in situ*) and bottom-up (*in vitro*) approaches were employed to reveal the mechanism of interaction between *A. pasteurianus* and *L. helveticus*, which are the two dominant bacteria in SSF of SAV. Their growth is negatively correlated to each other both *in situ* and *in vitro*, and there is no nutritional competition between them. The growth and metabolism of *L. helveticus* were inhibited due to the presence of *A. pasteurianus*, indicating an amensalism relationship between them. Ethanol, acetic acid, and lactic acid were proved the most important endogenous factors that regulate the growth profiles of *A. pasteurianus* and *L. helveticus*. Transcriptomic analysis results showed that in *L. helveticus* the genes in metabolic pathways of starch and sucrose, galactose, fatty acids and some amino acids were down-regulated under co-culture condition comparing to its mono-culture condition, while the genes in metabolic pathways of glycerophospholipid, energy (ABC transporters), pyrimidine and purine were up-regulated. The number of transcriptionally regulated genes was less in *A. pasteurianus* than in *L. helveticus*. The genes for KEGG pathways of sugar, amino acid, purine, and pyrimidine metabolism were up-regulated in *A. pasteurianus* under co-culture condition, and metabolic pathways of pyruvate, glyoxylate, fructose and mannose were down-regulated comparing to its mono-culture condition. These results prove the amensalism between *A. pasterurinaus* and *L. helveticus*. This work gives a whole insight on the interaction between the predominant species in SSF of cereal vinegar from nutrient utilization, endogenous factors inhibition and the regulation of gene transcription.

## Data availability statement

The datasets presented in this study can be found in online repositories. The names of the repository/repositories and accession number(s) can be found in the article/supplementary material.

## Author contributions

MX and XZ performed the experiments and substantially contributed to the acquisition, analysis, and interpretation of data. YX and QS were involved in the experiments and revised and discussed the manuscript. LT and FC were involved in revising the manuscript. YY was involved in the experiments. YZ and MW designed the study and were involved in drafting and revising the manuscript. All authors contributed to the article and approved the submitted version.

## Funding

This work was supported by the National Natural Science Foundation of China (32072203), the Tianjin Synthetic Biotechnology Innovation Capacity Improvement Project (TSBICIP-KJGG-016-03), the Tianjin Science and Technology Commission (21ZYJDJC00030), Key Research and Development Program of Ningxia (2022BBF02010), and the Shanxi Science and Technology Department (2022D100194051319014450459217).

## Conflict of interest

YY is employed by Shanxi Zilin Vinegar Industry Co., Ltd.

The remaining authors declare that the research was conducted in the absence of any commercial or financial relationships that could be construed as a potential conflict of interest.

## Publisher’s note

All claims expressed in this article are solely those of the authors and do not necessarily represent those of their affiliated organizations, or those of the publisher, the editors and the reviewers. Any product that may be evaluated in this article, or claim that may be made by its manufacturer, is not guaranteed or endorsed by the publisher.

## Footnotes

^1^ https://sourceforge.net/projects/metamis/

^2^ http://www.spss.com.hk/

^3^ http://www.genome.jp/kegg/
